# Peer Review of “Mask Use to Curtail Influenza in a Post–COVID-19 World: Modeling Study”

**DOI:** 10.2196/37240

**Published:** 2022-05-27

**Authors:** Julia Frederick

**Affiliations:** 1 Department of Environmental Health Science University of Georgia Athens, GA United States

**Keywords:** mask, protection, COVID-19, influenza, transmission, intervention, infectious disease, respiratory, simulation, model, prevalence, efficacy

*This is a peer-review report submitted for the paper “Mask Use to Curtail Influenza in a Post–COVID-19 World: Modeling Study.”*

## Round 1 Review

### General Comments

This paper [1] investigates how influenza cases can be decreased by the implementation of masks by a varying proportion of the population. It uses previous research and historical case rates by the Centers for Disease Control and Prevention (CDC) to form its model. Overall, this paper’s model is an informative look at a potential scenario for future flu seasons. As it calculates a lot of its variables from a data set, knowing what these values will be and how they compare with the current literature can make the reader’s confidence in the model stronger.

### Specific Comments

#### Major Comments

1. The final sentence of the Abstract needs to be completed or reworked to explain “other practical aspects.”

2. I’m assuming that this is all focused on solely the United States since it is using CDC data. However, noting that this is US-centric and giving a brief description of how the CDC acquires this data will help the reader understand the data set, especially with many of the CDC data sets being underrepresentative of actual case rates because they are highly dependent on medical reports. In the case of the flu, how many people get the flu but never report to the CDC or see a doctor to get treatment because symptoms are mild?

3. A creation of a table of or explicitly stating the variables and values used in the model is important for understanding. Especially when it comes to the calculated variables like B(t). Is that the same for each of those curves or is it changing with the different curves? If so, how much does it vary?

#### Minor Comments

4. How much does the virulence of the flu strains for that year versus the efficacy of the vaccine that year affect the data you are working with? Are there years that you think the masks would have helped substantially more than other years because the vaccine efficacy was lower than expected?

5. What is the typical mask efficacy for respiratory viruses? How does this “real-world” efficacy rate compare to the efficacy rates that you are using in your model?

## Abbreviations

CDC: Centers for Disease Control and Prevention
